# A Comprehensive Review on Understanding Magnesium Disorders: Pathophysiology, Clinical Manifestations, and Management Strategies

**DOI:** 10.7759/cureus.68385

**Published:** 2024-09-01

**Authors:** Manjeet Kothari, Anil Wanjari, Suhail M Shaikh, Parav Tantia, Bhavana V Waghmare, Avinash Parepalli, Khadija F Hamdulay, Manikanta Nelakuditi

**Affiliations:** 1 Internal Medicine, Jawaharlal Nehru Medical College, Datta Meghe Institute of Higher Education and Research, Wardha, IND; 2 Obstetrics and Gynaecology, Jawaharlal Nehru Medical College, Datta Meghe Institute of Higher Education and Research, Wardha, IND

**Keywords:** magnesium supplementation, electrolyte disorders, magnesium homeostasis, hypermagnesemia, hypomagnesemia, magnesium deficiency

## Abstract

Magnesium is vital in a broad spectrum of physiological processes, including enzyme activity, energy production, and neuromuscular function. Despite its crucial role, magnesium disorders - comprising both deficiency (hypomagnesemia) and excess (hypermagnesemia) - are frequently underrecognized and inadequately managed in clinical practice. Magnesium deficiency is widespread, particularly among populations with chronic illnesses, the elderly, and those experiencing malnutrition, often leading to significant neuromuscular, cardiovascular, and metabolic complications. Conversely, hypermagnesemia, though less common, poses serious risks, especially in individuals with impaired renal function or those receiving high doses of magnesium supplements or medications. This review comprehensively examines magnesium disorders, detailing their pathophysiology, clinical manifestations, and management strategies. It highlights the essential functions of magnesium in maintaining cellular integrity, cardiovascular health, and bone structure and discusses the global prevalence and risk factors associated with magnesium imbalances. By offering insights into the current understanding of magnesium homeostasis and its disruptions, this review aims to enhance the awareness and treatment of magnesium-related conditions, ultimately improving patient outcomes across diverse clinical settings.

## Introduction and background

Magnesium is an essential mineral that is critical in numerous physiological processes. Despite being the fourth most abundant cation in the human body and the second most prevalent intracellular cation, its importance is often underappreciated. Understanding magnesium’s functions, the impact of its dysregulation, and the prevalence of related disorders is vital for comprehensive patient care [1]. Magnesium is indispensable for a wide array of cellular functions. It is a cofactor for over 300 enzymatic reactions crucial for metabolizing carbohydrates, fats, and proteins. This includes ATP-dependent processes, where magnesium stabilizes ATP, the primary energy currency of the cell. Additionally, magnesium plays a significant role in DNA and RNA synthesis, protein synthesis, and maintaining the structural integrity of nucleic acids [2]. The mineral’s influence extends to the nervous system, which helps regulate neuromuscular function by modulating the activity of neurotransmitters and muscle contractions. Magnesium is essential in maintaining the electrical potential across cell membranes, contributing to normal nerve conduction and muscle function. In the cardiovascular system, magnesium aids in maintaining vascular tone and preventing arrhythmias by influencing calcium handling within heart cells. Furthermore, it plays a role in bone health, with about 60% of the body’s magnesium residing in bone tissue, which contributes to bone mineralization and strength [3].

Magnesium disorders, encompassing both deficiency (hypomagnesemia) and excess (hypermagnesemia), are relatively common but often underdiagnosed. Magnesium deficiency is more prevalent and can result from inadequate dietary intake, gastrointestinal losses, or renal excretion. Conditions such as chronic alcoholism, malabsorption syndromes, and prolonged use of diuretics contribute significantly to magnesium deficiency [4]. Magnesium deficiency varies globally, with higher rates observed in populations with limited access to magnesium-rich foods or those with chronic illnesses. The elderly are particularly at risk due to age-related changes in absorption and excretion, as well as the common use of medications that may deplete magnesium levels. Malnutrition, whether due to socioeconomic factors or disease states, further exacerbates the risk [5]. Hypermagnesemia, though less common, can occur in individuals with impaired renal function or in those receiving excessive magnesium through medications or supplements. It is particularly concerning in patients with chronic kidney disease, where the body's ability to excrete magnesium is compromised [6]. This review aims to provide a comprehensive understanding of magnesium disorders, exploring their pathophysiology, clinical manifestations, and management strategies. By highlighting the importance of magnesium in human physiology and the prevalence of related disorders, this review aims to underscore the need for increased awareness and better clinical management of these conditions.

## Review

Pathophysiology of magnesium homeostasis

Absorption and Distribution

Magnesium is primarily absorbed in the gastrointestinal tract, particularly in the small intestine, with the jejunum and ileum being key absorption sites. This process occurs through two primary mechanisms: passive paracellular and active transcellular transport. Several factors influence magnesium absorption, including dietary intake, other nutrients, and vitamin D levels. For example, higher dietary magnesium intake generally enhances absorption, while certain gastrointestinal conditions can hinder it [7]. Once absorbed, magnesium is distributed throughout the body, with approximately 99% residing in bone and soft tissues. Specifically, about 60% of magnesium is stored in the bones, while the remaining 40% is found in soft tissues, including muscles and organs. Only about 1% of the body's magnesium is present in extracellular fluid, which is critical in various physiological functions. The kidneys are essential in regulating magnesium levels, filtering and reabsorbing magnesium throughout the nephron. Approximately 20-30% of filtered magnesium is reabsorbed in the proximal tubule, 60-70% in the thick ascending limb of the loop of Henle, and 5-10% in the distal convoluted tubule. This renal regulation ensures magnesium balance by adjusting excretion according to the body’s needs [8].

Regulation of Magnesium Levels

**Figure 1:**
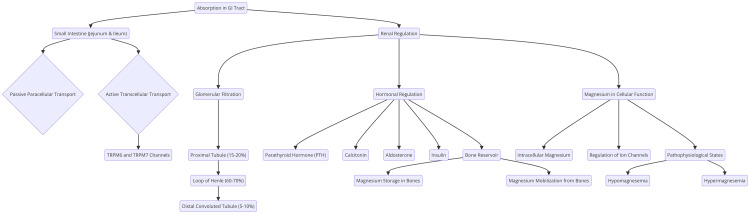
The pathophysiology of magnesium homeostasis Image credit: Dr. Manjit Kothari

Several hormonal and biochemical factors influence the regulation of magnesium levels. Parathyroid hormone (PTH) and calcitonin are key players in magnesium homeostasis. PTH enhances renal magnesium reabsorption, while calcitonin also promotes magnesium retention. Additionally, vitamin D increases the intestinal absorption of magnesium, demonstrating the interconnectedness of these nutrients in maintaining overall mineral balance [9]. Magnesium interacts with other electrolytes, particularly calcium and potassium. A magnesium deficiency can disrupt the balance of these electrolytes, potentially leading to complications such as hypocalcemia and hypokalemia. This interaction highlights the crucial role of magnesium in the broader context of electrolyte homeostasis [10]. Moreover, acid-base balance significantly affects magnesium homeostasis. In conditions of metabolic acidosis, the kidneys typically reduce magnesium reabsorption, resulting in increased urinary excretion. Conversely, metabolic alkalosis can enhance renal magnesium reabsorption, influencing overall magnesium levels [11]. The pathophysiology of magnesium homeostasis is illustrated in Figure 1.

Magnesium deficiency (hypomagnesemia)

Etiology

The etiology of hypomagnesemia is multifactorial, involving dietary, gastrointestinal, renal, and pharmacological factors. Inadequate dietary intake of magnesium-rich foods, such as leafy greens, nuts, seeds, and whole grains, can result in insufficient magnesium levels, especially in populations that rely heavily on processed foods. Certain life stages, such as pregnancy and lactation, also increase magnesium requirements, making adequate dietary intake even more critical [4]. Malabsorption syndromes, including celiac disease and Crohn's disease, can impair the small intestine's ability to absorb nutrients, including magnesium. Chronic pancreatitis can disrupt digestion and nutrient absorption, contributing to magnesium deficiency. Chronic diarrhea from conditions like irritable bowel syndrome (IBS), infections, or inflammatory bowel disease can cause significant magnesium loss, as magnesium is excreted in the stool [12]. Renal causes of hypomagnesemia include the use of diuretics, alcohol consumption, and genetic disorders. Medications such as loop diuretics (e.g., furosemide) and thiazide diuretics can increase urinary excretion of magnesium, leading to depletion. Chronic alcohol use can impair renal function and elevate magnesium excretion, further contributing to deficiency. Gitelman syndrome, a rare genetic disorder, affects renal tubular function, resulting in excessive loss of magnesium and other electrolytes, such as potassium and calcium, in the urine [13]. Several medications can also contribute to magnesium deficiency by increasing renal excretion or impairing absorption. Long-term use of proton pump inhibitors (PPIs), such as omeprazole and esomeprazole, has been associated with reduced magnesium absorption in the gut. Certain antibiotics, particularly aminoglycosides, can affect renal function and lower magnesium levels. Chemotherapy agents may also lead to electrolyte imbalances, including magnesium depletion [14].

Clinical Manifestations

Magnesium is an essential mineral crucial for various physiological processes. Magnesium deficiency, known as hypomagnesemia, can manifest in various clinical symptoms, primarily affecting the neuromuscular, cardiovascular, and metabolic systems [9]. Magnesium is vital for proper muscle function and nerve transmission. Low magnesium levels can result in various neuromuscular symptoms. Muscle cramps, characterized by involuntary contractions of the skeletal muscles, are common, particularly affecting the legs, feet, and hands. In more severe cases, hypomagnesemia can lower the seizure threshold, increasing the risk of seizures, especially in individuals with a history of epilepsy. Additionally, tremors may involve uncontrollable shaking of the hands, head, or other body parts. Tetany, marked by severe muscle spasms and contractions, particularly in the hands and feet, is one of the most serious manifestations and can lead to life-threatening complications if not addressed promptly [15]. Magnesium is crucial for maintaining normal cardiac function. Its deficiency can lead to various cardiovascular issues, including an increased risk of arrhythmias such as atrial fibrillation, ventricular tachycardia, or torsades de pointes. Low magnesium levels have also been associated with an elevated risk of developing hypertension, although the mechanisms behind this relationship remain unclear. Some studies suggest a potential link between magnesium deficiency and an increased risk of coronary artery disease, though further research is needed to confirm a definitive causal relationship [16]. Magnesium deficiency can lead to several adverse effects on metabolic processes. One significant consequence is the development of insulin resistance, which is associated with hypomagnesemia and an increased risk of type 2 diabetes. The exact mechanisms of this relationship are still being studied. Magnesium is also essential for the proper absorption and utilization of calcium; thus, low magnesium levels can result in hypocalcemia, manifesting as muscle cramps, seizures, and abnormal heart rhythms. Additionally, magnesium deficiency can impair the body’s ability to maintain normal potassium levels, leading to hypokalemia, which may cause muscle weakness, fatigue, and an increased risk of cardiac arrhythmias [17].

Diagnosis

Diagnosing hypomagnesemia requires a combination of laboratory tests and clinical evaluation due to magnesium's critical role in various physiological processes. Accurate diagnosis is essential to prevent complications associated with magnesium deficiency [18]. The primary diagnostic test for hypomagnesemia is the measurement of serum magnesium levels. Normal serum magnesium levels generally range from 1.3 to 2.1 mEq/L (0.65 to 1.05 mmol/L). Levels below 1.5 mg/dL (0.75 mmol/L) indicate hypomagnesemia. However, since most magnesium is stored in bones and tissues, serum magnesium levels may not fully reflect total body magnesium stores. Therefore, additional tests may be needed for a thorough assessment [19]. Ionized magnesium, the biologically active form, can be measured directly through specialized blood tests. This measurement may offer a more accurate reflection of magnesium status, particularly in patients with altered protein levels or acid-base imbalances. A 24-hour urine collection can also be useful to evaluate renal magnesium handling. Low urinary magnesium excretion suggests gastrointestinal loss or inadequate dietary intake. In contrast, high urinary magnesium may indicate renal loss, which can occur due to diuretics, certain endocrine disorders, or genetic conditions [20].

When diagnosing hypomagnesemia, it is crucial to consider other electrolyte disorders and conditions that may present with similar symptoms. Hypokalemia, which can occur alongside hypomagnesemia due to increased renal potassium wasting, should be evaluated since it can exacerbate magnesium deficiency. Therefore, serum potassium levels should be measured alongside magnesium levels. Additionally, hypocalcemia should be assessed as magnesium affects PTH secretion and action. Low magnesium can impair PTH function, leading to low calcium levels, making it important to evaluate serum calcium levels in patients with hypomagnesemia [21]. Primary hyperaldosteronism is another condition to consider, as it can cause both hypomagnesemia and hypokalemia due to increased renal excretion of these electrolytes. Plasma aldosterone and renin levels may be measured to assess for this condition. Furthermore, patients on diuretics often experience electrolyte imbalances, including hypomagnesemia, hypokalemia, and hyponatremia. A thorough medication history and electrolyte panel are essential in these cases [22]. Chronic kidney disease (CKD) is also a significant consideration, as it can alter magnesium metabolism, leading to either hypermagnesemia or hypomagnesemia, depending on the stage and treatment. Kidney function tests should be performed, such as serum creatinine and glomerular filtration rate. Lastly, gastrointestinal disorders like Crohn's disease or celiac disease can impair magnesium absorption. In such cases, stool studies and imaging may be necessary to assess gastrointestinal function [23].

Management strategies

Managing hypomagnesemia involves a comprehensive approach that includes oral magnesium supplementation, intravenous magnesium therapy, and addressing underlying causes contributing to the deficiency. Each strategy is essential for effective treatment and preventing recurrence [24].

Oral Magnesium Supplementation

Oral magnesium supplementation is typically the first line of treatment for mild to moderate hypomagnesemia. Magnesium supplements include magnesium oxide, citrate, chloride, and glycinate [25]. Magnesium oxide, which contains about 61% elemental magnesium, is commonly dosed at 400 mg daily, providing approximately 240 mg of elemental magnesium. Magnesium citrate, often better tolerated, provides about 48 mg of elemental magnesium per 5 mL solution. For optimal absorption and to minimize gastrointestinal side effects, these supplements should be taken with food [26]. The recommended daily allowance (RDA) for magnesium varies by age and sex: adult males require about 400-420 mg daily, while females need 310-360 mg daily. Regular monitoring of serum magnesium levels is crucial, especially in patients with ongoing gastrointestinal or renal issues [27].

Intravenous Magnesium Therapy

In cases of severe hypomagnesemia or when rapid correction is necessary, intravenous magnesium therapy may be indicated. This approach is particularly important for patients with cardiac arrhythmias, severe neuromuscular symptoms, or those unable to tolerate oral supplements due to gastrointestinal disturbances. An initial dose of 1-2 grams of magnesium sulfate can be administered over 15-30 minutes, with higher doses required for more severe cases [16]. A continuous infusion of 0.5 mmol/kg/day may be initiated for less urgent situations, with adjustments based on serum magnesium levels and patient response. Close monitoring is essential during IV therapy, including frequent checks of serum magnesium and other electrolytes, such as potassium and calcium, to prevent complications like hypermagnesemia. Healthcare providers should also be vigilant for signs of magnesium toxicity, such as bradycardia or respiratory depression, and be prepared to halt therapy if these symptoms arise [28]. Addressing the underlying causes of hypomagnesemia is critical for effective management. Malabsorption syndromes, including celiac disease or Crohn's disease, should be managed with appropriate dietary adjustments and potentially additional supplementation [9]. Reviewing the patient's medication regimen, such as diuretics or PPIs, is also important to identify and adjust medications that may promote magnesium loss. Alternatives or dose adjustments may be necessary. Encouraging patients to adopt a magnesium-rich diet, including nuts, seeds, whole grains, and green leafy vegetables, can help maintain adequate magnesium levels after treatment [9]. By integrating these management strategies, healthcare providers can effectively address hypomagnesemia, ensuring immediate deficiency correction and long-term maintenance of magnesium levels. This comprehensive approach is vital for improving patient outcomes and preventing potential complications associated with magnesium deficiency [29]. Management strategies for magnesium deficiency (hypomagnesemia) are shown in Table 1.

**Table 1:** Management strategies for magnesium deficiency (hypomagnesemia)

Management strategy	Description	Indications	Advantages	Challenges/considerations
Oral magnesium supplementation	Administration of magnesium salts (e.g., magnesium oxide, citrate, gluconate)	Mild to moderate hypomagnesemia; maintenance therapy	Non-invasive; easy to administer; cost-effective	GI side effects (e.g., diarrhea); variable absorption rates
Intravenous magnesium therapy	IV administration of magnesium sulfate	Severe hypomagnesemia; symptomatic patients; when rapid correction is needed	Rapid correction of magnesium levels; effective in severe cases	Requires monitoring of magnesium levels and renal function; potential for hypermagnesemia
Dietary modification	Increasing intake of magnesium-rich foods (e.g., green leafy vegetables, nuts, seeds, whole grains)	Mild hypomagnesemia; prevention in at-risk populations	Natural and holistic approach; provides additional nutrients	Dependent on patient compliance; limited effect in severe deficiency
Addressing underlying causes	Treating conditions contributing to magnesium loss (e.g., malabsorption syndromes, chronic diarrhea)	Chronic conditions causing hypomagnesemia	Addresses root cause of deficiency	May require multidisciplinary management; complex and time-consuming
Adjusting medications	Modifying or discontinuing medications that deplete magnesium (e.g., diuretics, proton pump inhibitors)	Medication-induced hypomagnesemia	Prevents further magnesium loss; improves overall electrolyte balance	Risk of compromising treatment of underlying conditions; requires careful assessment
Monitoring and follow-up	Regular monitoring of serum magnesium levels and clinical symptoms	All cases of hypomagnesemia; especially in patients with chronic conditions	Ensures effective management; early detection of complications	Requires regular healthcare visits; patient adherence needed

Magnesium excess (hypermagnesemia)

Etiology

Hypermagnesemia, characterized by elevated levels of magnesium in the blood, can result from various etiological factors. Understanding these causes is essential for accurate diagnosis and effective management [30]. A common cause of hypermagnesemia is excessive magnesium intake, particularly from dietary sources. High magnesium supplements, often used for benefits like muscle relaxation or improved sleep, can elevate serum magnesium levels, especially if not properly monitored [6]. Additionally, many over-the-counter antacids contain magnesium compounds, such as magnesium hydroxide, which can contribute to hypermagnesemia when used excessively or inappropriately. Similarly, magnesium-containing laxatives, such as magnesium citrate and magnesium sulfate, used for constipation relief, can lead to significant magnesium absorption and subsequent hypermagnesemia when overused [31]. Renal failure is another critical factor in the development of hypermagnesemia, as the kidneys are responsible for magnesium excretion. Acute kidney injury (AKI) can result in a sudden loss of kidney function, leading to decreased magnesium excretion and elevated serum magnesium levels. Causes of AKI include dehydration, sepsis, and nephrotoxic drugs. CKD also significantly contributes to hypermagnesemia, as the kidneys progressively lose their ability to filter waste products, including excess magnesium, particularly in the later stages of the disease [6]. Other factors contributing to hypermagnesemia include adrenal insufficiency and hypothyroidism. Conditions such as Addison's disease can disrupt electrolyte balance due to insufficient adrenal hormone production, leading to elevated magnesium levels. Similarly, hypothyroidism, characterized by low thyroid hormone levels, can impair renal function and reduce magnesium excretion [32]. Certain medications, especially those containing magnesium, can also induce hypermagnesemia. For example, magnesium sulfate treats eclampsia, asthma, and certain cardiac conditions. However, excessive or prolonged use can lead to hypermagnesemia. Other magnesium-containing drugs, such as some antacids and laxatives, can also contribute to elevated magnesium levels when used inappropriately [32].

Clinical Manifestations

Hypermagnesemia, characterized by elevated magnesium levels in the blood, can lead to various clinical manifestations affecting multiple body systems, particularly the neuromuscular, cardiovascular, and central nervous systems. Recognizing these symptoms is essential for early diagnosis and effective management of this electrolyte imbalance [33]. Neuromuscular symptoms are often the initial signs of hypermagnesemia. Patients may experience lethargy, which manifests as profound fatigue and reduced energy levels, impeding their ability to perform daily activities. Muscle weakness is another prevalent symptom, affecting both proximal and distal muscle groups and leading to difficulties in movement and coordination. Additionally, a reduction in deep tendon reflexes is frequently observed during physical examinations, indicating neuromuscular impairment. In severe cases, hypermagnesemia can result in respiratory depression, where paralysis of the respiratory muscles leads to shallow breathing or respiratory failure, a critical condition requiring immediate medical attention [34]. The cardiovascular system is highly sensitive to changes in magnesium levels, and hypermagnesemia can lead to several serious complications. Hypotension, or low blood pressure, is a common cardiovascular manifestation where elevated magnesium levels cause vasodilation. This can result in symptoms such as dizziness or fainting, especially upon standing. Bradycardia, characterized by a slower-than-normal heart rate, may also occur and can be detected on an electrocardiogram (ECG) or through clinical assessment [35]. Hypermagnesemia can also lead to various cardiac arrhythmias, including atrial fibrillation, marked by an irregular and rapid heart rate and complete heart block, where electrical signals are completely obstructed, causing significant bradycardia. In extreme cases, particularly when serum magnesium levels exceed 7 mmol/L, the risk of cardiac arrest increases due to severe disruptions in cardiac conduction [16]. The central nervous system can also be significantly affected by hypermagnesemia. Patients may exhibit altered mental status, including confusion and disorientation, which can complicate diagnosis as it may be mistaken for other neurological conditions. In severe instances, hypermagnesemia can progress to coma, characterized by unresponsiveness and a lack of protective reflexes, representing a medical emergency that demands immediate intervention [33].

Diagnosis

Diagnosing hypermagnesemia involves a comprehensive approach that includes laboratory tests, clinical evaluation, and, occasionally, imaging studies. The primary diagnostic test is the measurement of serum magnesium levels. Normal serum magnesium concentrations typically range from 1.7 to 2.2 mg/dL (0.7 to 1.0 mmol/L). Serum magnesium levels above 2.6 mg/dL (1.1 mmol/L) indicate hypermagnesemia, while levels exceeding 4.0 mg/dL (1.65 mmol/L) are considered severe. It is crucial to interpret elevated magnesium levels with clinical symptoms and other laboratory findings to obtain a complete clinical picture [36]. In addition to serum magnesium levels, a comprehensive metabolic panel (CMP) is essential for assessing overall electrolyte balance. This panel measures sodium, potassium, calcium, and bicarbonate, helping to identify any coexisting electrolyte disturbances that may occur alongside hypermagnesemia, such as hypocalcemia or hyperkalemia [37]. Renal function tests, including blood urea nitrogen (BUN) and creatinine levels, are also vital, as impaired renal function is a common cause of hypermagnesemia. In some cases, measuring urinary magnesium excretion can provide additional insight; low urinary excretion might suggest a renal cause, whereas high excretion could indicate excessive intake or redistribution [37]. Imaging studies may be employed to investigate the underlying causes of hypermagnesemia further. A kidney ultrasound can evaluate renal anatomy and function, particularly in patients with suspected AKI or CKD. This imaging can reveal structural abnormalities contributing to renal impairment and subsequent hypermagnesemia. An ECG may also be used to monitor for cardiac arrhythmias or conduction disturbances associated with hypermagnesemia, such as bradycardia or prolonged PR intervals [38]. If an endocrine cause is suspected, additional laboratory tests may be necessary. For example, thyroid function tests, including thyroid-stimulating hormone (TSH) and free T4, can assess thyroid health. Serum cortisol levels may be measured if adrenal insufficiency is a concern. A thorough diagnostic approach that integrates serum magnesium measurement, electrolyte assessment, renal function evaluation, and targeted imaging studies is essential for accurately diagnosing hypermagnesemia and guiding appropriate management strategies [39].

Management Strategies

Immediately discontinuing magnesium-containing products is the initial and most critical step in managing hypermagnesemia. This includes various medications and supplements, such as antacids, laxatives, magnesium-based enemas, and multivitamins containing magnesium. The primary goal of this intervention is to halt further increases in serum magnesium levels by eliminating the source of excess magnesium. This step is especially important for patients who may have been using these products for conditions such as gastrointestinal discomfort or constipation, particularly if they have underlying renal impairment that affects magnesium excretion [6]. In cases of hypermagnesemia, intravenous calcium administration is an effective countermeasure against the physiological effects of elevated magnesium levels. Magnesium and calcium share similar physiological pathways, and elevated magnesium can lead to neuromuscular and cardiovascular disturbances, including bradycardia and hypotension [33]. Administering calcium gluconate or chloride helps stabilize cardiac membranes and restore normal neuromuscular function. A common protocol involves administering 10-20 mL of a 10% calcium gluconate solution over five to 10 minutes. This intervention is particularly useful in acute settings for the rapid reversal of symptoms, providing essential support while other treatments take effect [33]. For patients with severe hypermagnesemia, especially those with compromised renal function, additional therapeutic measures may be necessary. Loop diuretics, such as furosemide, can enhance renal magnesium excretion. However, caution is required when using diuretics in patients with renal impairment, as they may exacerbate fluid and electrolyte imbalances [40]. Dialysis may be needed when hypermagnesemia is life-threatening or when other treatments are insufficient for magnesium clearance. Hemodialysis or continuous renal replacement therapy (CRRT) can efficiently remove excess magnesium from the bloodstream, providing a rapid and effective means of correction. This approach is crucial for patients with end-stage renal disease, or AKI, where the kidneys cannot adequately excrete magnesium [40]. Ongoing monitoring and supportive care are essential in managing hypermagnesemia. Regular assessment of serum magnesium levels is crucial to evaluate the effectiveness of treatment and guide further management decisions. Levels should be checked every two to four hours until they stabilize within the normal range [41]. Additionally, monitoring other electrolytes, particularly calcium and potassium, is important, as imbalances can complicate the clinical picture and exacerbate symptoms. Supportive care, including maintaining adequate hydration and monitoring vital signs, is critical in ensuring patient safety and comfort. By providing comprehensive care that includes vigilant monitoring and supportive interventions, healthcare providers can effectively manage hypermagnesemia and minimize its potential complications [41]. Management strategies for magnesium excess (hypermagnesemia) are shown in Table 2.

**Table 2:** Management strategies for magnesium excess (hypermagnesemia)

Management strategy	Description	Indications	Key considerations
Discontinuation of magnesium sources	Ceasing intake of magnesium-containing supplements, medications, or antacids.	Initial approach for mild to moderate hypermagnesemia.	Important to identify all potential sources of magnesium.
Intravenous calcium administration	Administration of calcium gluconate or calcium chloride intravenously to counteract the effects of magnesium.	Symptomatic hypermagnesemia (e.g., cardiovascular or neuromuscular symptoms).	Acts as an antagonist to magnesium, helping to stabilize cardiac and neuromuscular functions.
Diuretics (loop diuretics)	Use loop diuretics (e.g., furosemide) to enhance renal excretion of magnesium.	Hypermagnesemia in patients with adequate renal function.	Monitor electrolyte levels to prevent hypokalemia and other imbalances.
Dialysis (hemodialysis or peritoneal dialysis)	Dialysis to remove excess magnesium from the bloodstream, especially in patients with renal failure.	Severe hypermagnesemia, particularly in patients with impaired renal function.	Hemodialysis is more effective than peritoneal dialysis in rapidly reducing magnesium levels.
Supportive care and monitoring	Continuous monitoring of vital signs, cardiac function, and electrolyte levels.	All cases, particularly moderate to severe hypermagnesemia.	Ensure close cardiac rhythms and respiratory status monitoring, especially in symptomatic patients.

Magnesium disorders in special populations

Magnesium disorders can significantly affect specific populations, including pregnant women, children, and the elderly, each presenting unique challenges related to magnesium's role, deficiency, and management [42]. Magnesium is critical during pregnancy because it involves various physiological processes, such as cellular function, muscle contraction, nucleic acids, and protein synthesis. Pregnant women require increased magnesium to support fetal development and placental function and to prevent complications like preeclampsia and eclampsia. Magnesium sulfate is commonly used in managing preeclampsia and eclampsia, serving as a neuroprotective agent for the fetus and helping to prevent seizures in the mother [43]. Magnesium deficiency during pregnancy has been associated with adverse outcomes, including preeclampsia, fetal growth restriction, and preterm labor. Maternal magnesium deficiency can result in restricted fetal growth and a higher risk of complications such as intrauterine growth restriction (IUGR) and preterm birth. Animal studies suggest that maternal hypomagnesemia may impair placental development and fetal growth, potentially leading to long-term consequences like metabolic syndrome. Epidemiological studies also indicate that low magnesium levels are correlated with increased risks of gestational diabetes and hypertensive disorders during pregnancy [44]. Magnesium is equally important for the growth and development of infants and children. It plays a crucial role in bone health, contributing to the structural development of bones and teeth, as well as in energy production and muscle function. Pediatric magnesium disorders can stem from factors such as dietary insufficiency, gastrointestinal issues, and congenital disorders. Management usually involves dietary adjustments to include magnesium-rich foods (e.g., nuts, seeds, whole grains) and, in some cases, supplementation under medical supervision. Regular monitoring of magnesium levels is essential, particularly for at-risk populations [45]. In older adults, changes in magnesium metabolism, such as decreased intestinal absorption and altered renal excretion, can lead to a higher prevalence of magnesium deficiency. These issues are often exacerbated by reduced dietary intake and chronic illnesses [46]. Additionally, older adults frequently face comorbidities and are often prescribed multiple medications that can impact magnesium balance. Certain medications, including diuretics and PPIs, may increase magnesium loss or interfere with absorption. Effective management for this population involves regular monitoring of magnesium levels, dietary counseling to ensure adequate intake, and careful evaluation of medications to minimize the risk of deficiency or excess [46].

Emerging research and future directions

Recent research advances the development of new magnesium compounds and formulations to enhance their therapeutic potential. One promising area involves the creation of magnesium chelates, which combine magnesium with amino acids or organic acids to improve absorption and minimize gastrointestinal side effects [47]. Another innovative approach uses magnesium liposomes, encapsulating magnesium in lipid-based carriers to enhance targeted delivery and reduce renal excretion. Additionally, researchers are exploring magnesium nanoparticles, which can enhance magnesium supplements' solubility, stability, and permeability, potentially leading to improved bioavailability and clinical efficacy [47]. There is also growing interest in understanding magnesium's role in chronic diseases. Studies suggest that magnesium may protect cardiovascular health by reducing inflammation, improving endothelial function, and lowering blood pressure, which could potentially decrease cardiovascular risk. Magnesium's involvement in metabolic processes is also being investigated, particularly its effects on glucose homeostasis and insulin sensitivity, with potential implications for diabetes prevention and management [48]. Emerging evidence indicates magnesium levels could be important biomarkers for disease prognosis and management. In cardiovascular health, low magnesium levels have been linked to an increased risk of hypertension, atherosclerosis, and adverse cardiovascular events [49]. Magnesium deficiency has also been associated with neurological disorders such as migraines, depression, and Alzheimer's disease, suggesting that monitoring magnesium levels could provide valuable insights into these conditions. In metabolic disorders, hypomagnesemia is commonly observed in individuals with type 2 diabetes and may contribute to the development of diabetic complications. As research progresses, magnesium may become a critical biomarker for assessing disease risk and guiding treatment strategies [49]. Addressing global magnesium deficiency requires comprehensive public health strategies. Updating dietary guidelines to emphasize the importance of magnesium-rich foods, such as whole grains, nuts, seeds, and leafy greens, is crucial. Food fortification initiatives could also increase the magnesium content of commonly consumed products, enhancing overall dietary intake. Targeted supplementation programs for high-risk populations, including the elderly, pregnant women, and individuals with chronic diseases, may also be necessary to ensure adequate magnesium levels [50]. Promoting adequate magnesium intake through updated dietary guidelines and targeted supplementation could have significant public health implications. Ensuring sufficient magnesium levels may help reduce the risk of chronic diseases such as cardiovascular conditions, metabolic disorders, and neurological issues. Furthermore, optimizing magnesium status could enhance the effectiveness of treatments for various health conditions, including hypertension, arrhythmias, and asthma [51]. Given its low cost and favorable safety profile, magnesium supplementation represents a valuable public health strategy with the potential to improve patient outcomes and reduce the burden of chronic diseases on healthcare systems globally. By focusing on emerging research and public health initiatives, we can advance our understanding of magnesium's role in health and disease, ultimately leading to better health outcomes for at-risk populations [51]. Emerging research and future directions in magnesium disorders are shown in Table 3.

**Table 3:** Emerging research and future directions in magnesium disorders

Area of research	Focus	Key findings/goals	Potential impact
Novel therapeutic approaches	Development of new magnesium compounds and formulations	Investigation of bioavailability and effectiveness of new magnesium salts and chelates.	Improved treatment outcomes for magnesium deficiency and potential new therapeutic options.
Magnesium in chronic diseases	Exploring the role of magnesium in chronic diseases such as cardiovascular and metabolic disorders	Studies link magnesium levels to preventing and managing hypertension, diabetes, and heart disease.	Enhanced understanding of magnesium’s role in chronic disease management and potential preventive strategies.
Magnesium as a biomarker	Evaluating magnesium levels as biomarkers for disease prognosis and management	Research into magnesium as a predictive marker for disease progression and response to therapy.	Potential for personalized medicine approaches based on magnesium status.
Public health strategies	Addressing global magnesium deficiency through dietary guidelines and supplementation programs	Exploration of fortification of foods with magnesium and public health campaigns.	Reduction in the prevalence of magnesium deficiency-related conditions at the population level.
Magnesium and neurodegenerative diseases	Investigating the role of magnesium in neuroprotection and cognitive health	Ongoing studies on magnesium’s potential to prevent or mitigate neurodegenerative diseases like Alzheimer’s and Parkinson’s.	Development of new neuroprotective strategies and potential therapeutic applications.
Magnesium and pregnancy outcomes	Researching the impact of magnesium on maternal and fetal health	Studies focus on magnesium’s role in preventing preeclampsia and supporting fetal development.	Improved maternal and neonatal health outcomes.

## Conclusions

In conclusion, magnesium is essential for various physiological processes, including cellular function, neuromuscular activity, cardiovascular health, and bone integrity. However, magnesium disorders - both deficiency and excess - are often overlooked in clinical practice despite their significant health impacts. Hypomagnesemia is particularly common among the elderly, those with chronic illnesses, and individuals with poor nutrition, highlighting the need for careful monitoring and management. Hypermagnesemia, although rarer, poses serious risks, especially in patients with renal impairment. This review emphasizes the importance of understanding magnesium homeostasis, recognizing the clinical manifestations of its dysregulation, and implementing effective management strategies. By doing so, healthcare professionals can improve patient outcomes through timely detection, intervention, and prevention of magnesium-related disorders.
